# Mediastinal tumor resection in a patient with spinocerebellar degeneration

**DOI:** 10.1186/s13019-020-01218-8

**Published:** 2020-07-29

**Authors:** Eiyu Tsuboi, Yoko Azuma, Takashi Makino, Takashi Terada, Hajime Otsuka, Atsushi Sano, Satoshi Koezuka, Takashi Sakai, Naobumi Tochigi, Akira Iyoda

**Affiliations:** 1 Division of Chest Surgery, Department of Surgery, Toho University School of Medicine, 6-11-1 Omori-nishi, Ota-ku, Tokyo, 143-8541 Japan; 2 Department of Anesthesiology, Toho University School of Medicine, Tokyo, Japan; 3 Department of Surgical Pathology, Toho University School of Medicine, Tokyo, Japan

**Keywords:** Surgery, Mediastinal tumor, Spinocerebellar degeneration

## Abstract

**Background:**

In spinocerebellar degeneration (SCD) patients, general and regional anesthesia may cause postoperative dysfunction of respiratory, nerve and muscle systems. We present the surgical case of thymoma developed in patient with SCD.

**Case presentation:**

A 47-year-old woman with spinocerebellar degeneration was admitted because of a well-defined mass measuring 48 × 31 mm in anterior mediastinum. She showed limb, truncal, ocular, and speech ataxia; hypotonia; areflexia; sensory disturbances; and muscle weakness. Her eastern cooperative oncology group performance status was 4. Surgical resection was performed via video-assisted thoracic surgery and under general anesthesia only without epidural analgesia. The mass was diagnosed as type B1 thymoma without capsular invasion (Masaoka stage I). The patients got a good postoperative course by cooperation with anesthesiologists and neurologists in perioperative managements. She has been well over 3 years of follow-up.

**Conclusions:**

In conclusion, careful surgical and anesthesia management is essential for providing an uneventful postoperative course in patients with SCD. Especially, selection of minimal invasive approach and avoid diaphragmatic nerve damage are the most important points in surgical procedures.

## Background

Spinocerebellar degeneration (SCD) or spinocerebellar ataxia (SCA) refers to a group of hereditary ataxias that are progressive, manifesting as degenerative changes of various parts of the central nervous system. The cerebellum, cerebral cortex, inferior olivary nucleus, basal ganglia, substantia nigra, and the spinal cord are involved. For patients with SCD requiring regional or general anesthesia, several issues regarding anesthetic management have been described [1], although reports on the use of video-assisted thoracoscopic surgery (VATS) are rare [2]. Here, we describe the surgical management of a patient with thymoma and severe SCD.

## Case presentation

**Fig. 1:**
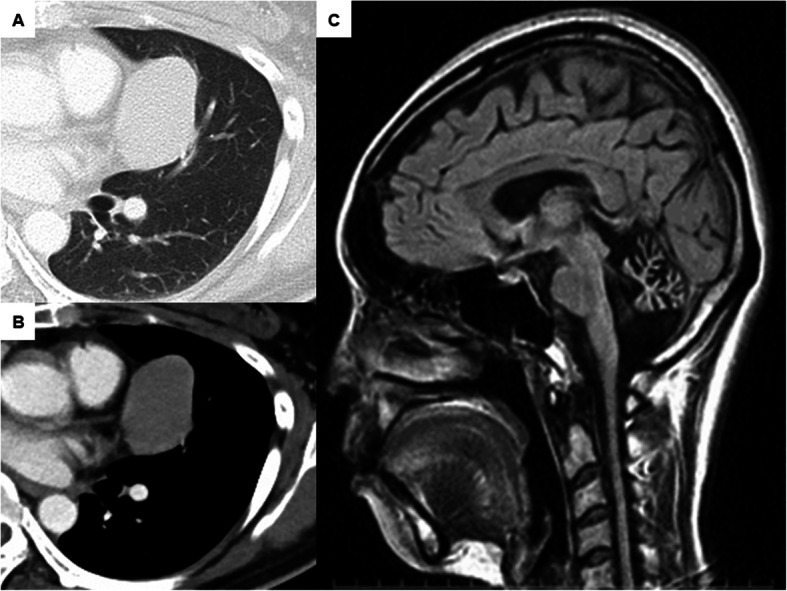
Imaging findings of the patents. Chest computed tomography shows (**a**) lung window image and (**b**) mediastinal window image of a well defined 48-mm mass in the left anterior mediastinum, in contact with pericardium and left lung. Sagittal weighted magnetic resonance image shows(**c**) severe cerebellar atrophy in a patient with spinocerebellar degeneration

A 47-year-old woman was admitted to our hospital because of an abnormal chest shadow found on a routine chest X-ray. SCD was diagnosed when she was 6 years of age. Chest computed tomography showed a well defined mass (48 × 31 mm) touching the pericardium and left lung (Fig. 1a and b). Laboratory examinations showed normal serum levels of alpha fetoprotein (2.4 ng/mL), human chorionic gonadotropin beta (< 0.2 ng/mL), and antiacetylcholine receptor antibody (< 0.2 nmol/L). The differential diagnosis included thymoma, thymic carcinoma, and germ cell tumor; and surgical resection was recommended. However, the patient was a high-risk surgical patient because of SCD. Physical examination revealed a patient who was 164 cm tall, weighing 56 kg. Her vital signs were normal. Neurological examination revealed limb, truncal, ocular, and ataxic dysarthria; hypotonia; areflexia; sensory disturbances; and muscle weakness. Her Eastern cooperative oncology group performance status was 4. Pulmonary function tests showed an obstructive pattern. Her vital capacity (1.57 L) was 56.3% of predicted value and her forced expiratory volume in 1 s /forced vital capacity was 70.5% of predicted value. Magnetic resonance imaging showed severe cerebellar atrophy and spinocerebellar degeneration (Fig. 1c).

We decided to follow the patient while evaluating her general condition. Three months after her initial diagnosis, her tumor had grown to 50 × 35 mm. We performed surgery with the patient under general anesthesia only (without epidural analgesia), after explaining the risk of respiratory failure in detail and obtaining consent from her and her family. She received 30 mg rocuronium bromide (0.5 mg/kg), target-controlled propofol intravenous infusion (4.0 μg/mL), and remifentanil intravenous infusion (0.2 μg/kg/min) as general anesthesia by single-lung ventilation via a double-lumen endotracheal tube. Resection of the mediastinal tumor was performed via VATS. Although the tumor was firmly adherent to the left phrenic nerve, the tumor was carefully resected to preserve the nerve.

**Fig. 2:**
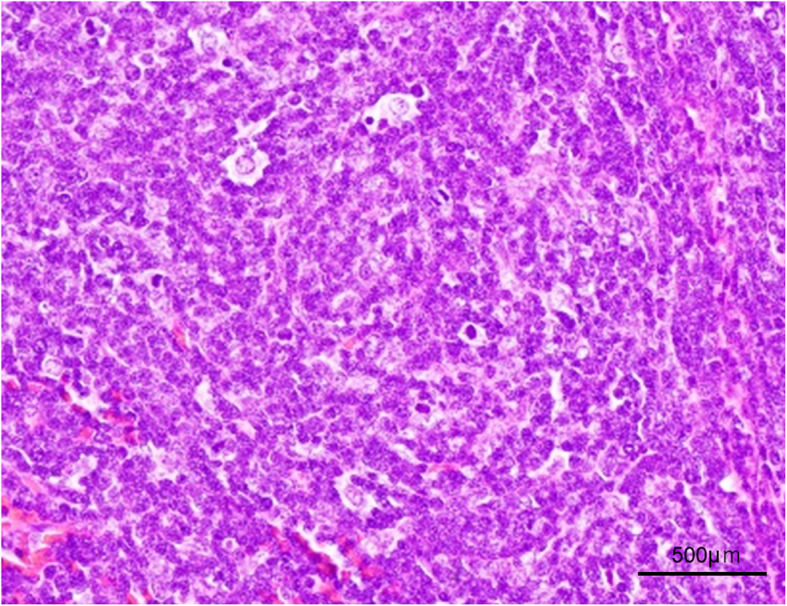
Pathological findings of the tumor. Photomicrograph showing small lymphocytes and middle-sized atypical epithelium-like thymic cells, suggesting type B1 thymoma (hematoxylin and eosin staining)

Histopathological examination of the tumor revealed small lymphocytes and atypical thymic cells of intermediate size that resembled epithelial cells (Fig. 2). Immunohistochemical staining showed that the small lymphocytes were positive for CD99 expression and the medium-sized atypical cells were positive for cytokeratin AE1/AE3 and negative for c-kit and CD5 expression. The lesion was diagnosed as type B1 thymoma without capsular invasion (Masaoka stage I). The patient’s postoperative course was uneventful, and she was discharged from the hospital on postoperative day 9. At the time of this report, 36 months after resection, she was doing well.

## Discussion

SCD refers to a group of hereditary progressive neurodegenerative diseases. The more than 30 types are classified according to the mutated gene associated with the specific SCD. There are degenerative changes in the cerebellum, brain stem, basal ganglia, pyramidal tract, somatosensory spinal pathways, optic nerve, and peripheral nerves. Degeneration in the cerebellum, which controls movement, leads to ataxia. There are many other signs and symptoms that reflect the anatomical sites of SCD-related lesions, which include signs of Parkinsonism, facial palsy, mental deterioration, disorders of speech and swallowing, muscle weakness involving the limbs and trunk, hypotonia, tremors, and ocular disturbances (nystagmus, double vision) and/or retinal degeneration [3, 4].

Currently no effective treatments are available for SCD, and care remains supportive [5]. In addition, SCD patients needing surgery are at risk of perioperative complications secondary to manifestations of their disease; therefore, careful surgical and perioperative management of these patients is very important.

Thoracic surgery for the patient with SCD is extremely rare. Yamauchi et al. [2] previously reported on VATS resection of 2 simultaneous mediastinal cysts (thymic cyst in anterior mediastinum and thoracic duct cyst in posterior) in a 60-year-old patient with SCD. The patient’s postoperative course was uneventful. However, the authors did not report details on the type of SCD or perioperative management. In our patient, resection of the mediastinal tumor was performed via VATS, and care was taken to prevent injury to the left phrenic nerve. Injury of the phrenic nerve leads to dysfunctional respiratory muscles; therefore protection of the phrenic nerve is essential, especially in a patient with SCD. VATS approach may provide minimally invasive operation but also clear visibility to avoid nerve injury.

Previous reports on the successful management of SCD patients under general and regional anesthesia have indicated that there are several problems, as follows [1, 6, 7]: [1] intra-and postoperative respiratory complications such as aspiration and respiratory muscle dysfunction, [2] signs of progression of neurological problems due to epidural anesthesia, [3] hyperkalemia due to depolarizing relaxants, [4] change in sensitivity to depolarizing muscle relaxant, [5] possible muscle rigidity due to fentanyl, and [6] extreme hemodynamic changes and changes in body temperature associated with injury to the sympathetic nervous system.

Anesthesia-related problems for patients with SCD include the development of respiratory complications. Respiratory muscle dysfunction leads to aspiration and postoperative pneumonia. The muscle relaxant for a patient with spinocerebellar atrophy must be chosen carefully because of the increased risk associated with prolonged neuromuscular paralysis and mechanical ventilation [8]. The use in our case of rocuronium bromide, which is a rapidly acting aminosteroid and is a nondepolarizing neuromuscular blocker, was associated with uneventful postoperative course without the development of respiratory muscle dysfunction.

## Conclusions

In conclusion, resection of a mediastinal tumor in a patient with SCD is very rare. Careful management of patients with SCD under general anesthesia is essential for providing an uneventful postoperative course. Finally, protection of the phrenic nerve in thoracic surgery is especially important for the patient with SCD. VATS approach may be suitable for SCD patients regarding both minimal invasion and good visibility.

## Data Availability

The data supporting the conclusions of this article are included within the article.

## Abbreviations

SCD: Spinocerebellar degeneration
SCA: Spinocerebellar ataxia
VATS: Video-assisted thoracoscopic surgery
